# Synthesis, photocatalytic and antidiabetic properties of ZnO/PVA nanoparticles

**DOI:** 10.1038/s41598-021-90846-8

**Published:** 2021-06-01

**Authors:** Shady M. EL-Dafrawy, Mahmoud Tarek, Salem Samra, Shawky M. Hassan

**Affiliations:** Chemistry Department, Faculty of Science, Mansoura University, Mansoura, Egypt

**Keywords:** Biochemistry, Biological techniques, Drug discovery, Environmental sciences, Chemistry, Nanoscience and technology

## Abstract

A series of ZnO and ZnO/poly(vinyl alcohol) (PVA) catalysts were prepared using sol–gel method. An X-ray diffraction analysis confirmed the existence of the wurtzite ZnO phase, and scanning electron microscopy (SEM) observation revealed the formation of spherical ZnO and ZnO/PVA nanoparticles. The decomposition of methylene blue (MB) and methyl orange (MO) induced by the synthesized pure ZnO and ZnO/PVA nanoparticles was studied under ultraviolet–visible irradiation. Among the catalysts evaluated, ZnO/5PVA was the most active in the decomposition of MB, whereas ZnO/7PVA was the most active catalyst in the decomposition of MO. Moreover, an investigation of the biological activity of pure ZnO and ZnO/PVA indicated that ZnO/5PVA exhibited the best performance in lowering the glucose level in diabetic rats.

## Introduction

Nanocomposites are a special type of materials with unique properties and a wide range of applications in diverse areas^1–4^. With the development of nanotechnology, nanopowders have emerged as a powerful tool for combining features of parent constituents in a single material, thereby obtaining nanocomposites with novel properties. Recently, ZnO, which is a well-known and important semiconductor, has received increasing research attention because of its wide potential applications in the photocatalytic oxidation of organic compounds^5^, solar cells^6^, and sensors^7^. In particular, the photocatalytic activity of ZnO, along with that of TiO_2_, in the degradation of organic pollutants in water and air has been the focus of research because of the unique ability of these metal oxides in environmental detoxification^8–15^. ZnO, which exhibits a bandgap of 3.37 eV, has proved to be the most suitable in photocatalysis because of its high stability and photosensitivity and wider absorption range in the solar spectrum compared with that of TiO_2_^16^.

Many chemical and physical synthesis methods have been investigated with the aim of improving the photocatalytic activity of ZnO nanoparticles^17–19^. In this study, an ecofriendly and affordable ZnO photocatalyst was synthesized using the sol–gel method. The synthesized samples were calcined at various temperatures, and their characterization was performed using several techniques. The synthesized ZnO catalyst was used to investigate the removal of methyl orange (MO) and methylene blue (MB) dyes as well-known water pollutants with adverse health effects for humans and animals^20^. In addition, the biological activity of ZnO in diabetic rats was evaluated.

Aiming to improve the photocatalytic and biological properties of ZnO, poly(vinyl alcohol) (PVA) was selected as a matrix and loaded onto the ZnO surface because of its very unique and significant physical and mechanical characteristics. For instance, PVA possesses high optical transparency, dielectric strength, solubility in water, nontoxicity, biodegradability, biocompatibility, excellent film-forming properties, good gas barrier properties against ambient gases, and doping-dependent electrical and optical properties, which arise from the presence of OH groups that form hydrogen bonds^21,22^.

## Experimental

### Materials

Zn acetate dihydrate (Zn(CO_2_CH_3_)_2_·2H_2_O, 98%), oxalic acid (H_2_C_2_O_4_, 99.5%), and ethanol (C_2_H_6_O, 99%) of analytical grade were used without purification. MB and MO were purchased from Aldrich Chemicals. PVA was used as a surface modifier (Sigma Aldrich, Mw = 86,000 g/mol). Double distilled water was used to prepare aqueous solutions.

### Preparation of the catalysts

#### Preparation of ZnO nanoparticles

The ZnO nanoparticles were prepared using the sol–gel method. Initially, a solution of 10.99 g Zn(CO_2_CH_3_)_2_·2H_2_O in 300 mL EtOH was stirred vigorously for 1 h. In another beaker, 17.71 g H_2_C_2_O_4_ was mixed with 200 mL ethanol, stirred for 1 h at 50 °C, and added slowly to the previous solution. A white gelatinous precipitate was obtained, which was dried under vacuum at 90 °C for 2 h and then calcined at different temperatures (300 °C, 400 °C, and 500 °C).

#### Preparation of ZnO/PVA nanoparticles

**Figure 1:**
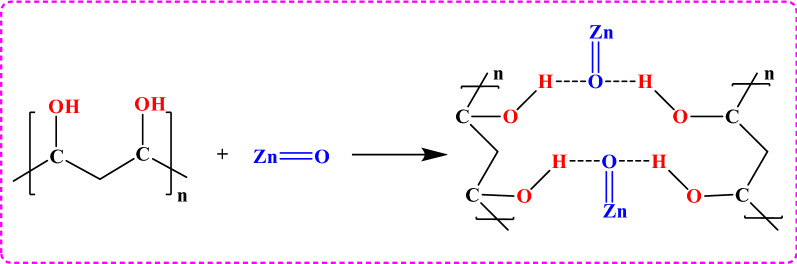
Reaction scheme of ZnO/PVA nanocomposites.

To prepare 5%, 7%, and 10% ZnO/PVA nanoparticles, a solution of PVA (0.25, 0.35, and 0.5 g, respectively) in 35 mL ethanol was added to a Zn(CO_2_CH_3_)_2_·2H_2_O solution (10.99 g in 300 mL ethanol) as presented in Fig. 1, and a precipitate was obtained using a H_2_C_2_O_4_ solution, as mentioned in Sect. 2.2.1. The product was obtained as a powder after vacuum drying followed by calcination at the same temperatures mentioned earlier.

### Characterization

#### X-ray diffraction

X-ray diffraction (XRD) patterns were recorded on a PW 150 diffractometer (Philips) using Ni-filtered Cu Kα radiation in a 2θ range of 10°–80° to investigate the formation of ZnO, the ZnO/PVA nanoparticle phases, and the crystal size in a nondestructive manner^23^.

#### Ultraviolet–visible spectrometry

The wavelength and absorbance values of the prepared photocatalysts obtained using an ultraviolet–visible (UV–Vis) reflectance spectrophotometer was utilized to determine the bandgap values from Kubelka–Munk equation^24,25^.

#### Scanning electron microscopy

The sizes of the ZnO and ZnO/PVA particles were determined using scanning electron microscopy (SEM) analysis on the JSM-5900 LV microscope with an accelerating voltage of 20 kV^26^.

#### Fourier transform infrared spectroscopy

The Brönsted and Lewis acid sites of ZnO and ZnO/PVA were investigated through Fourier transform infrared spectroscopy (FTIR) analysis on a Bruker spectrophotometer.

#### Potentiometric titration

Nonaqueous potentiometric titration was used to determine the total number of acid sites of the samples^27^. First, 0.1 g of a catalyst was activated by heating under vacuum for 2 h. Then, it was immersed into 10 mL acetonitrile for 3 h to be adsorbed on the active sites, followed by the titration of this suspension against a 0.01 N *n*-butyl amine solution (0.05 mL/min). An Orion 420 digital model device was utilized to determine the variation of the electrode potential.

#### The MB and MO degradation in water using UV–Vis light irradiation

The photocatalytic activities of the ZnO and ZnO/PVA nanoparticles were estimated by conducting degradation experiments at 20 °C using an external lamp (Halogen Mercury lamp, 400 W UV/Vis lamps).

#### Antidiabetic activity

Before the experiment, 12 albino rats (100–120 g) were kept for adaptation under normal laboratory conditions for 7 days. All rats were fed on a balanced basal diet and allowed free access of water.

##### Induced of diabetes mellitus

The experimental was then induced on the rats, which were divided randomly into two major groups:

**Group 1** (control diabetic rats): Four rats received a normal diet for 30 days without any treatment.

**Group 2**: Eight rats were fasted for 24 h and then intraperitoneally injected with streptozocin freshly prepared in a 0.1 M citrate buffer (pH 4.5) at a dose of 4.5 mg/100 g of body weight to induce diabetes mellitus according to a reported method^28^. Then, the rats were fasted for 18 h before determination of the serum glucose level. Rats with serum glucose levels over 250 mg/dL were considered as streptozocin diabetic rats and ready for treatment with ZnO and ZnO/PVA.

The eight diabetic rats of Group 2 were divided randomly into two series (four rats each) as follows:

**Series 1**, with rats treated with ZnO, ZnO/5PVA, ZnO/7PVA, and ZnO/10PVA with a dose of 3.5 mg/kg of body weight.

**Series 2**, with rats treated with ZnO, ZnO/5PVA, ZnO/7PVA, and ZnO/10PVA with a dose of 7 mg/kg of body weight.

Heparinized tubes with blood samples were collected from the eye canthus of the rats every 5 days after starting the administration of the extracts. Then, each blood sample was centrifuged to obtain a clear serum and determine immediately glucose levels of fasting animals.

- All experiments were performed in agreement with regulations of the Institutional Animal Ethics Committee of Mansoura University, Mansoura, Egypt, which are in accordance with the “Guide for the Care and Use of Laboratory Animals” published by the National Academy of Sciences.
- The study was conducted in compliance with the ARRIVE guidelines.

## Results and discussions

### X-ray analysis

**Figure 2:**
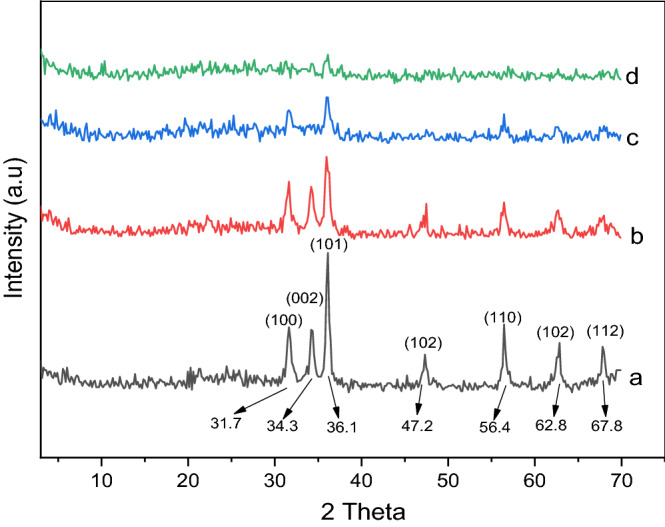
XRD pattern for (**a**) ZnO, (**b**) ZnO/5PVA, (**c**) ZnO/7PVA and (**d**) ZnO/10PVA calcined at 400 °C.

**Figure 3:**
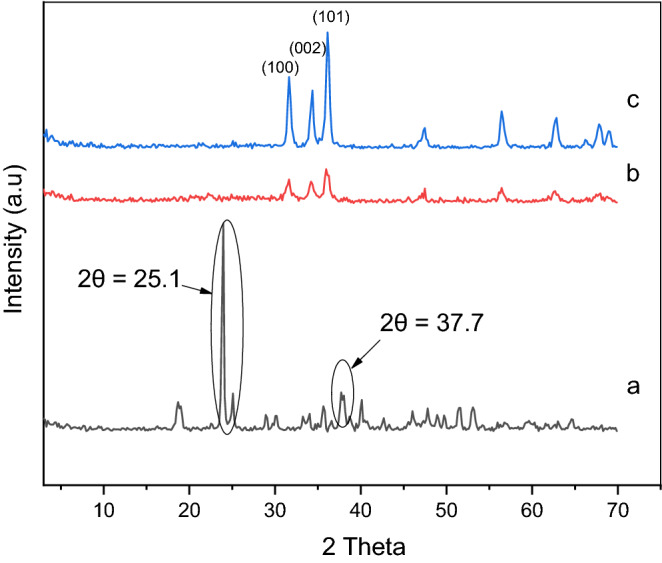
XRD pattern for ZnO/5PVA at calcination (**a**) 300, (**b**) 400 and (**c**) 500 °C.

The effect of the calcination temperature and the PVA concentration on the crystal phase and the crystal size of the synthetized samples was determined on the basis of the XRD patterns (Figs. 2 and 3). All samples exhibited typical hexagonal structures (JCPDS card no. 36–1451)^29^, and no phases attributable to PVA were observed. As can be seen in Fig. 2, the intensity of the (101), (002), and (100) peaks at 2θ = 36.1°, 34.3°, and 31.6°, respectively, decreased with increasing PVA content as a result of the increase in the crystal deformation. The (101) ZnO peak at 2θ = 36.1° was used to measure the crystal size of the ZnO/PVA samples. By increasing the quantity of PVA from 35–18 to 21–18 nm, a decrease in the crystallinity and the crystal size of ZnO was observed. In addition, increasing the PVA content led to a decrease in the mean grain size because PVA covered the crystalline surface of ZnO, causing a decrease in the diffraction intensity. Subsequently, further reduction in the crystal size occurred as the PVA substitution with Zn^2+^ ions increased^30,31^. Figure 3 indicates that at 300 °C, a peak appearing at 2θ = 25.1° can be assigned to Zn oxalate hydrate with a monoclinic structure, and that at 2θ = 37.7° corresponds to Zn oxalate with a monoclinic structure. By increasing the calcination temperature of ZnO/5PVA to 400 °C and 500 °C, the intensity of the (101), (002), and (100) peaks at 2θ = 36.1°, 34.3°, and 31.6°, respectively, corresponding to an hexagonal structure increased because the PVA content decreased upon the increase of temperature.

### UV–Vis absorption spectroscopy

**Figure 4:**
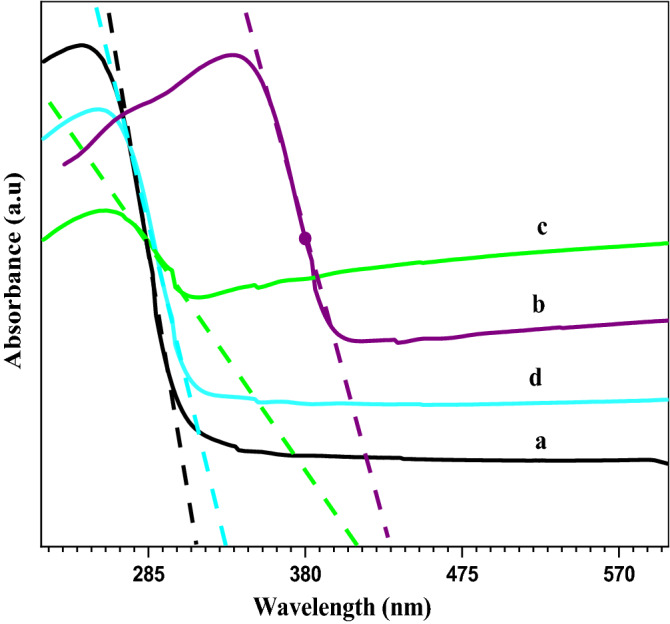
Band gab for (**a**) ZnO, (**b**) ZnO/5PVA, (**c**) ZnO/7PVA and (**d**) ZnO/10PVA calcined at 300 °C.

Figure 4 illustrates the variation of the bandgap energy. The absorbance values were introduced in the Kubelka–Munk equation to obtain (αhv)^2^ values^32^. Extrapolation of the linear region of (αhν)^2^ versus hν for the ZnO/PVA films indicated that the bandgap of the nanocomposites decreased slightly with the increasing quantity of ZnO in ZnO/PVA. The bandgap energy was determined to be 3.25 and 4.04 eV for ZnO and the unfilled PVA film, respectively. Meanwhile, the bandgap energy of the ZnO/PVA nanocomposite films ranged from 3.95 to 3.2 eV. The decrease in the bandgap energy caused a red shift in the absorption peak with the increase in the ZnO amount. This decrease in the bandgap energy may be due to the formation of a charge-transfer complex from the trap levels between the HOMO and LUMO energy states of PVA, which promotes lower energy transitions leading to the observed change in the bandgap^7^. The Eg values of ZnO and ZnO/PVA were 3.27 and 3.55 eV, respectively. The values of the bandgap energy for the samples containing PVA were 3.10–3.16 eV, which were smaller than those of pure oxides.

### SEM analysis

**Figure 5:**
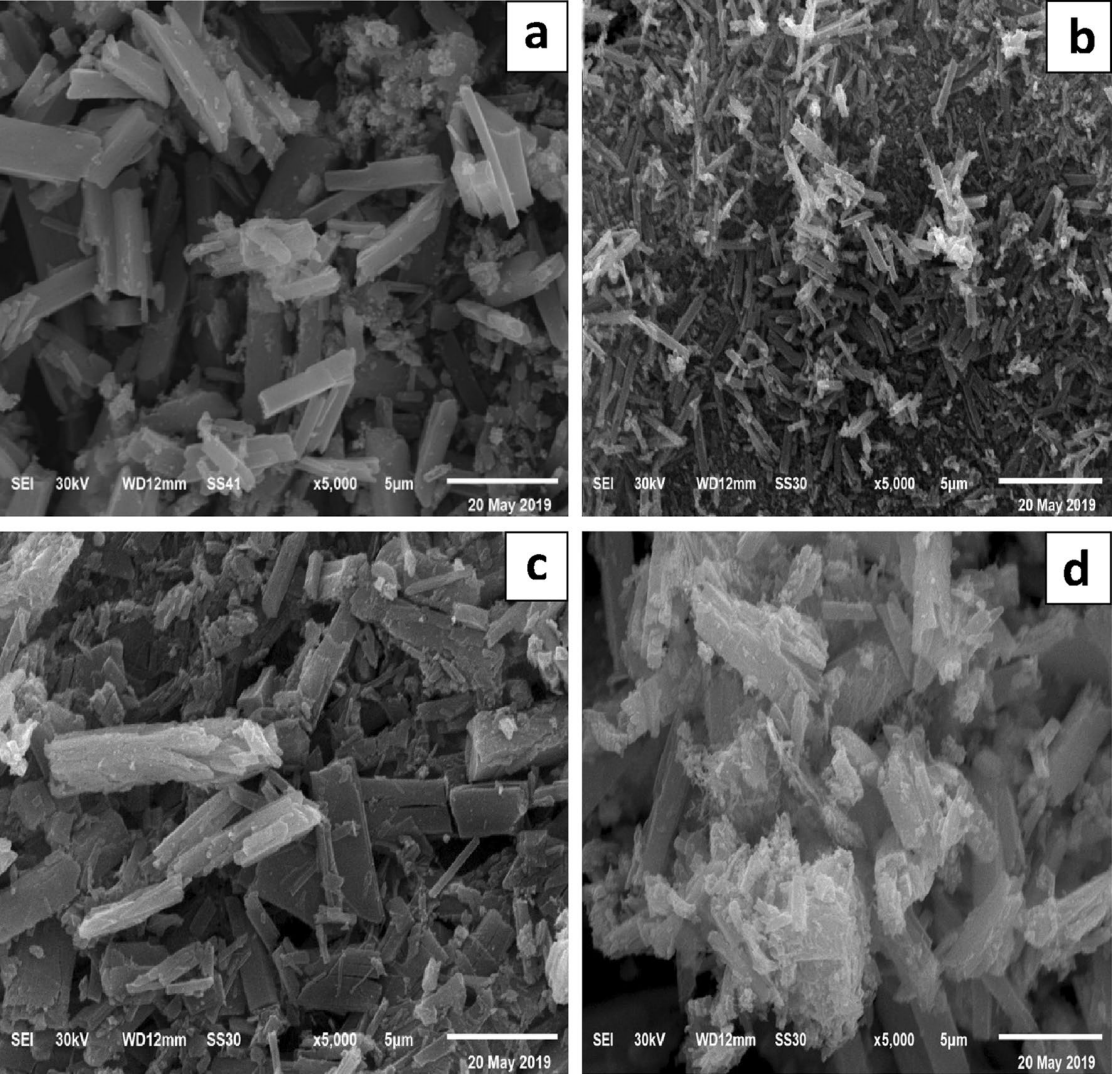
SEM images for (**a**) ZnO, (**b**) ZnO/5PVA, (**c**) ZnO/7PVA and (**d**) ZnO/10PVA calcined at 400 °C.

Figure 5 displays SEM images of selected samples of ZnO and ZnO/PVA photocatalysts with various PVA contents (5%, 7%, and 10%), in which agglomeration can be observed for all the samples to some extent. This particle aggregation may lead to the difference observed in the particle size obtained through the XRD and SEM analyses for ZnO and ZnO/PVA^33^. The SEM images revealed that the hexagonal phase increased with increasing PVA content. Small crystals with various phases were interwoven with each other, creating strongly bound nanoclusters^34^.

SEM was also used to confirm the distribution and size of ZnO in the polymeric matrix. ZnO appeared as white spots. It is obvious that the ZnO particles were well spread in the polymeric matrix regardless of the type of ZnO. This revealed that good adhesion between the surface of the ZnO nanoparticles and the polymeric matrix was achieved by modifying the organic surface of the ZnO nanoparticles.

### Acidity test

**Figure 6:**
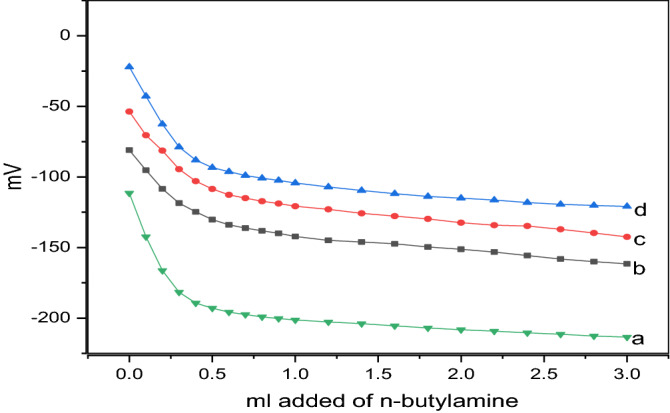
Acidity curve for (**a**) ZnO, (**b**) ZnO/5PVA, (**c**) ZnO/7PVA and (**d**) ZnO/10PVA calcined at 400 °C.

Potentiometric titration was utilized to estimate the number of surface acid sites of solid catalysts^35,36^. Neutralization of the surface acid sites was conducted by adding *n*-butylamine, and the electrode potential (mV) was evaluated as a function of the increasing concentration of *n*-butylamine (mmol/g catalyst)^37^. As can be extracted from Fig. 6, when the PVA content was increased up to 10%, the total number of acid sites increased. The amount of *n*-butyl amine/g used for the neutralization of the surface acid sites of the solid catalysts as a function of the PVA content at 500 °C and the total number of acid sites/g are summarized in Table 1.

**Table 1:** PVA content, Volume of n-butyl amine/g and total number of acid sites/g.

Sample	ml added	Total № of acid sites/g
ZnO	0.31	1.867 × 10^21^
ZnO/5PVA	0.42	2.529 × 10^21^
ZnO/7PVA	0.49	2.951 × 10^21^
ZnO/10PVA	0.92	5.541 × 10^21^

The total number of acid sites of the solid catalysts/g was determined using the following equation:

$$ {\text{Total}}\;{\text{number}}\;{\text{of}}\;{\text{acid}}\;{\text{sites/g}} = \left( {{\text{mL}}\;{\text{equivalent/g}}} \right) \times {\text{N}} \times 1000, $$

N, Avogadro's number.

### Photocatalytic activity

Next, the photocatalytic activity of the prepared ZnO and ZnO/PVA catalysts for the MB and MO degradation under irradiation with UV–Vis light was evaluated^38^.

First, a dry and clean bottle containing a 50 mL dye solution (10 ppm) and a 0.1 mg/L photocatalyst solution was stirred for 30 min in the dark. Subsequently, the bottle was irradiated with UV–Vis light for a certain period of time. An aliquot of 1 mL was taken out, diluted 10 times, and centrifuged for 15 min to settle down the catalyst. The dye concentration was measured using a UV–Vis spectrophotometer at a certain wavelength according to the λ_max_ of each dye (666 nm for MB and 464 nm for MO). Then, the percentage of removal was evaluated according to the following equation:

$$ \% \;removal = \frac{{c_{{\text{o}}} - c_{{\text{t}}} }}{{c_{{\text{o}}} }}*100, $$

C_0_: initial concentration of dye (mg/L) and C_t_: concentration of dye (mg/L) at time t (min).

#### Effect of the PVA concentration on the MB degradation

**Figure 7:**
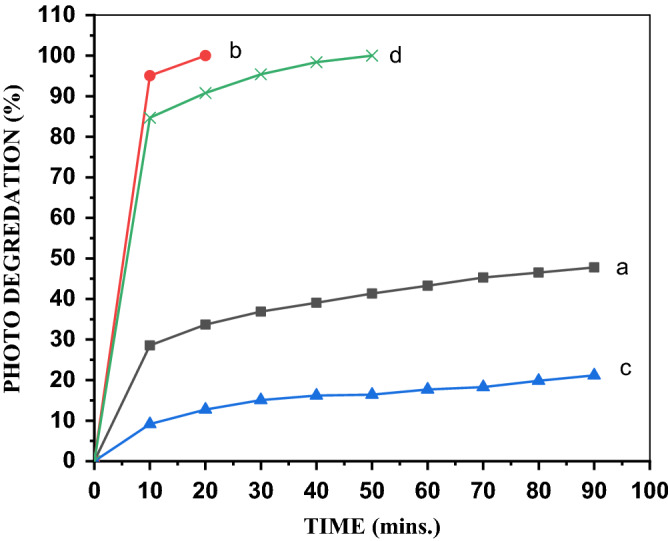
Photodegredation of (**a**) ZnO, (**b**) ZnO/5PVA, (**c**) ZnO/7PVA and (**d**) ZnO/10PVA calcined at 300 °C of MB.

Doping is an effective method of improving the activity of a wide-bandgap photocatalyst such as ZnO. The results summarized in Table 1 reveal that ZnO exhibited lower photocatalytic activity and higher number of acid sites than those of ZnO/PVA. For the MB cationic dye, the low photocatalytic activity of ZnO was probably due to (1) fast recombination of electrons and holes and (2) participation of a small part of these electrons and holes in the photocatalytic reaction^39^. Among the ZnO/PVA catalysts, ZnO/5PVA proved to have the optimum doping level leading to the maximum photocatalyst activity, which stems from the influence of PVA in decreasing the recombination rate and creating a new energy level in ZnO^24^. The photocatalytic activity decreased with the increase in the PVA content beyond 5%, as illustrated in Fig. 7. This is due to the high PVA content increasing the electron–hole recombination^40,41^.

#### Effect of the calcination temperature on the MB degradation

**Figure 8:**
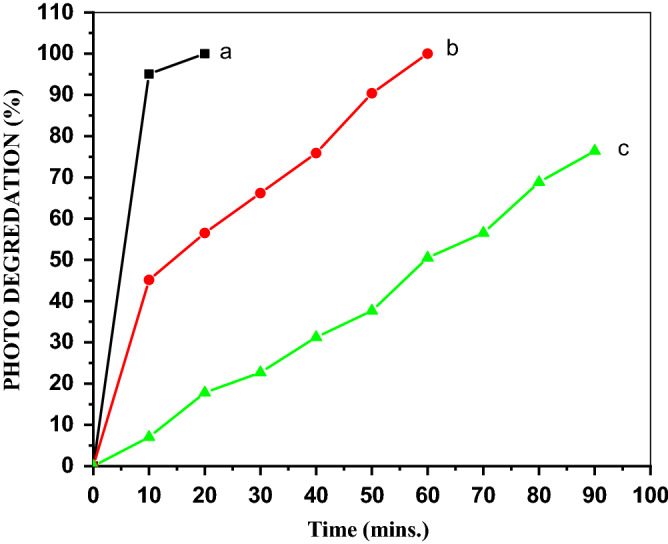
Photo degredation of MB by ZnO/5PVA calcined at (**a**) 300, (**b**) 400 and (**c**) 500 °C.

The SEM, XRD, and UV–Vis analyses proved that the temperature of calcination has a significant effect on the bandgap and structure of the catalysts, eventually affecting the photocatalytic performance. Figure 8 reveals the photocatalytic activity of ZnO/5PVA for the photodegradation of the MB cationic dye at calcination temperatures of 300 °C, 400 °C, and 500 °C. The ZnO/5PVA nanocomposite exhibited the best photocatalyst activity at a calcination temperature of 300 °C, as confirmed by the SEM and XRD results.

#### Effect of PVA concentration on MO degradation

**Figure 9:**
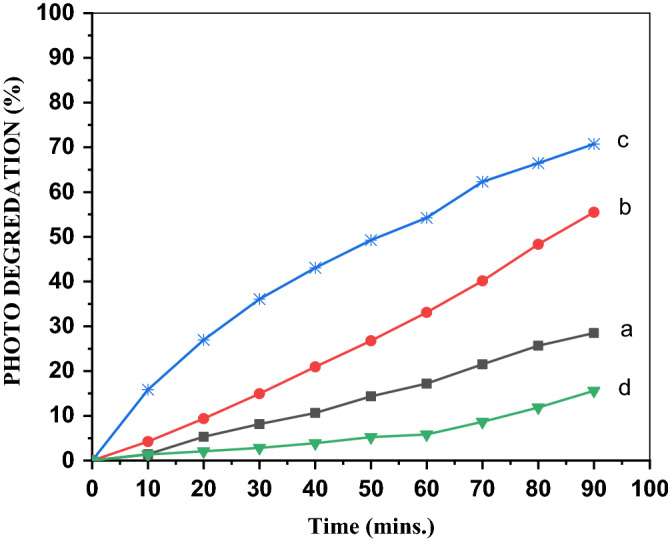
Photo degredation for (**a**) ZnO, (**b**) ZnO/5PVA, (**c**) ZnO/7PVA and (**d**) ZnO/10PVA calcined at 300 °C of MO.

Figure 9 indicates the activity of the different catalysts prepared from ZnO and diverse PVA concentrations calcined at 300 °C for the degradation of the MO anionic dye. Among the ZnO/PVA nanocomposites, ZnO/7PVA exhibited the optimum doping level to obtain the maximum photocatalytic activity. It was also found that the photocatalytic activity decreased upon increasing the PVA content beyond 7%.

#### Effect of the calcination temperature on the MO degradation

**Figure 10:**
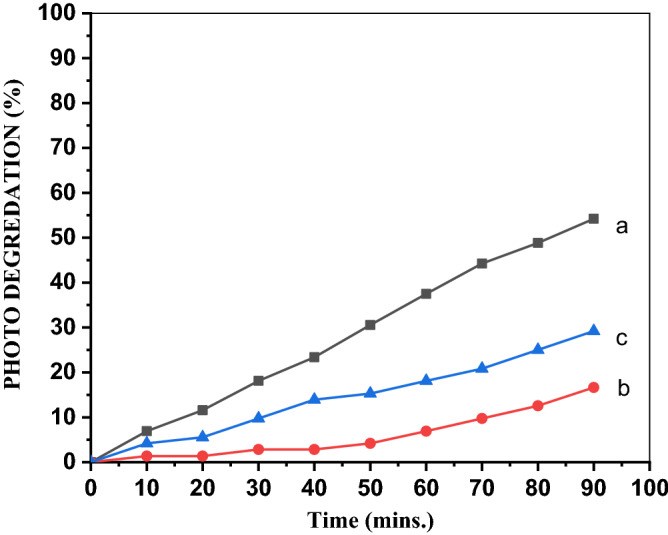
Photo degredation of MO by ZnO/7PVA calcined at (**a**) 300, (**b**) 400 and (**c**) 500 °C.

Figure 10 reveals that ZnO/7PVA calcined at 300 °C exhibited the best photocatalytic activity for photodegradation of the MO anionic dye among the photocatalysts evaluated.

## Biological activity

The effect of the catalysts on the blood glucose levels of the two series of rats described in Sect. 2.5 was evaluated considering the following factors^28^.

### Blood glucose level and the number of days at a constant PVA content

**Figure 11:**
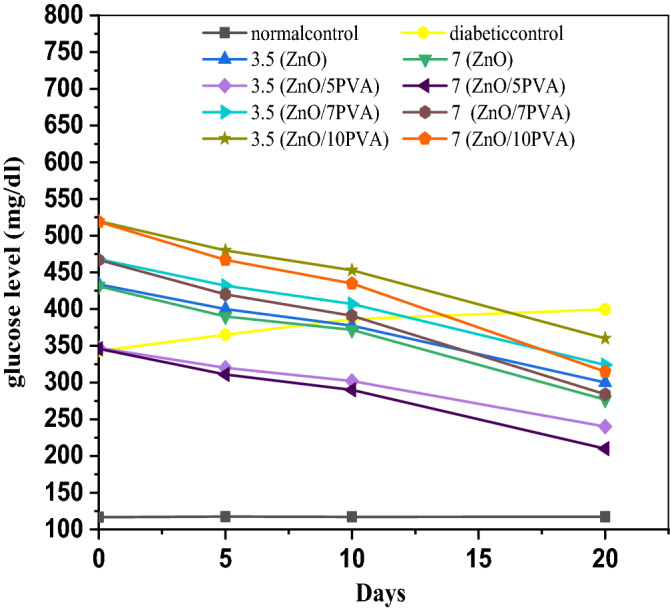
Glucose level against days for ZnO, ZnO/5PVA, ZnO/7PVA and ZnO/10PVA calcined at 300 °C for two series 3.5 mg and 7 mg.

The results summarized in Fig. 11 and Table 2 reveal that the lowest glucose level was achieved by the ZnO/5PVA catalyst calcined at 300 °C for the two series.

**Table 2:** Serum glucose level at different days and % of PVA for two series.

Levels of	Treatment period	Normal control	Diabetic control	ZnO dose	
				3.5 mg	7 mg
Serum Glucose at ZnO	Zero time	116.59 ± 03	343.11 ± 08	433.75	431.24
	5 days	117.44 ± 53	365.19 ± 04	400	390
	10 days	117.07 ± 01	386.35 ± 11	377.5	371.71
	20 days	117.21 ± 00	399.63 ± 00	300.01	276.62
Serum Glucose at ZnO/5PVA	Zero time	116.59 ± 03	343.11 ± 08	347.15	346.00
	5 days	117.44 ± 53	365.19 ± 04	320.00	311.65
	10 days	117.07 ± 01	386.35 ± 11	302.21	290.09
	20 days	117.21 ± 00	399.63 ± 00	240.00	210.88
Serum Glucose at ZnO/7PVA	Zero time	116.59 ± 03	343.11 ± 08	468.6	467.5
	5 days	117.44 ± 53	365.19 ± 04	432.2	420.7
	10 days	117.07 ± 01	386.35 ± 11	407.9	391.5
	20 days	117.21 ± 00	399.63 ± 00	324.1	284.6
Serum Glucose at ZnO/10PVA	Zero time	116.59 ± 03	343.11 ± 08	520.5	519.4
	5 days	117.44 ± 53	365.19 ± 04	480	467.3
	10 days	117.07 ± 01	386.35 ± 11	453.6	435
	20 days	117.21 ± 00	399.63 ± 00	360	315

### Blood glucose level and PVA content at a fixed number of days

**Figure 12:**
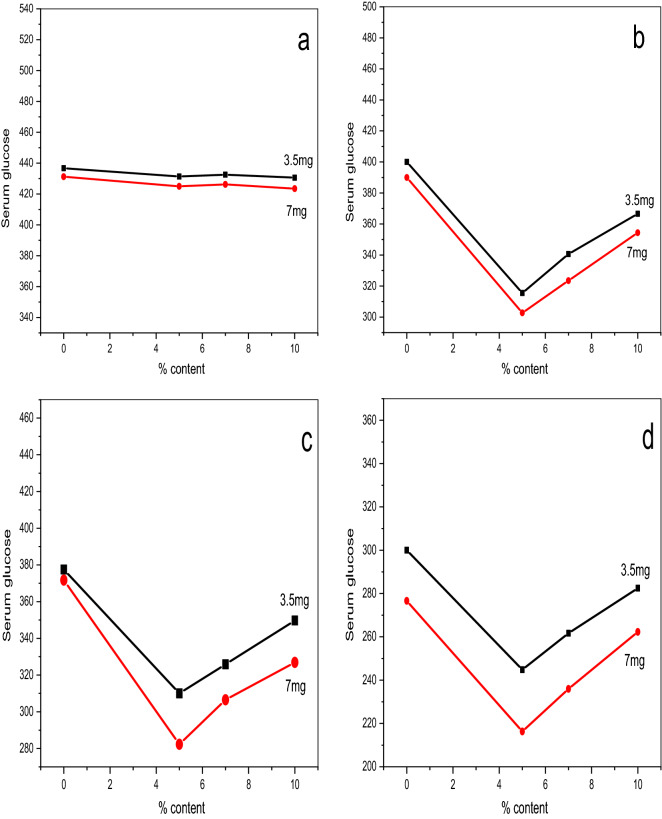
Glucose level against PVA content for (**a**) 0, (**b**) 5, (**c**) 10 and (**d**) 20 days calcined at 300 °C for two series 3.5 mg and 7 mg.

Figure 12 reveals that ZnO/5PVA afforded the lowest glucose level after 20 days for the two series.

## Conclusion

ZnO and ZnO/PVA nanoparticles were prepared using the sol–gel method. The formation of the hexagonal phase of polycrystalline ZnO and ZnO/PVA was confirmed by an XRD analysis. Meanwhile, an SEM analysis indicated that the ZnO and ZnO/PVA nanocomposites have a spherical shape. Photocatalytic experiments indicated that ZnO/5PVA is the most photocatalytically active catalyst for degradation of the MB cationic dye, whereas ZnO/7PVA is the most active for degradation of the MO anionic dye. The best results were obtained at a calcination temperature of 300 °C. Potentiometric titration proved that ZnO/10PVA had the highest number of total acid sites. As revealed by a biological activity study, ZnO/5PVA calcined at 300 °C was the best catalyst for decreasing the glucose level in diabetic rats. Therefore, it can be concluded that loading PVA onto the surface of ZnO improved the photocatalytic and biological properties of pure ZnO.
